# A Cecropin-4 Derived Peptide C18 Inhibits *Candida albicans* by Disturbing Mitochondrial Function

**DOI:** 10.3389/fmicb.2022.872322

**Published:** 2022-04-19

**Authors:** Chao-Qin Sun, Jian Peng, Long-Bing Yang, Zheng-Long Jiao, Luo-Xiong Zhou, Ru-Yu Tao, Li-Juan Zhu, Zhu-Qing Tian, Ming-Jiao Huang, Guo Guo

**Affiliations:** ^1^The Key and Characteristic Laboratory of Modern Pathogen Biology, School of Basic Medical Sciences, Guizhou Medical University, Guiyang, China; ^2^Key Laboratory of Environmental Pollution Monitoring and Disease Control, Guizhou Medical University, Ministry of Education, Guiyang, China; ^3^Translational Medicine Research Center, Guizhou Medical University, Guiyang, China; ^4^Center of Laboratory Medicine, The Affiliated Hospital of Guizhou Medical University, Guiyang, China; ^5^Department of Laboratory Medicine, The Second Affiliated Hospital of Guizhou Medical University, Kaili, China

**Keywords:** cecropin-4 derived peptide, *Candida albicans*, ROS, mitochondrial dysfunction, *G. mellonella*, antifungal activity

## Abstract

Global burden of fungal infections and related health risk has accelerated at an incredible pace, and multidrug resistance emergency aggravates the need for the development of new effective strategies. *Candida albicans* is clinically the most ubiquitous pathogenic fungus that leads to high incidence and mortality in immunocompromised patients. Antimicrobial peptides (AMPs), in this context, represent promising alternatives having potential to be exploited for improving human health. In our previous studies, a Cecropin-4-derived peptide named C18 was found to possess a broader antibacterial spectrum after modification and exhibit significant antifungal activity against *C. albicans*. In this study, C18 shows antifungal activity against *C. albicans* or non-*albicans Candida* species with a minimum inhibitory concentration (MIC) at 4∼32 μg/ml, and clinical isolates of fluconazole (FLZ)-resistance C. *tropicalis* were highly susceptible to C18 with MIC value of 8 or 16 μg/ml. Additionally, C18 is superior to FLZ for killing planktonic *C. albicans* from inhibitory and killing kinetic curves. Moreover, C18 could attenuate the virulence of *C. albicans*, which includes damaging the cell structure, retarding hyphae transition, and inhibiting biofilm formation. Intriguingly, in the *Galleria mellonella* model with *C. albicans* infection, C18 could improve the survival rate of *G. mellonella* larvae to 70% and reduce *C. albicans* load from 5.01 × 10^7^ to 5.62 × 10^4^ CFU. For mechanistic action of C18, the level of reactive oxygen species (ROS) generation and cytosolic Ca^2 +^ increased in the presence of C18, which is closely associated with mitochondrial dysfunction. Meanwhile, mitochondrial membrane potential (△Ψm) loss and ATP depletion of *C. albicans* occurred with the treatment of C18. We hypothesized that C18 might inhibit *C. albicans* via triggering mitochondrial dysfunction driven by ROS generation and Ca^2 +^ accumulation. Our observation provides a basis for future research to explore the antifungal strategies and presents C18 as an attractive therapeutic candidate to be developed to treat candidiasis.

## Introduction

In the past few years, the occurrence of systemic, life-threatening fungal infections caused by *Candida* spp. has increased dramatically, especially in immunocompromised patients (Houšt’ et al., 2020). *Candida albicans* (*C. albicans*), the predominant fungi, has been reported to account for as high as 35-50% of mortality rate in candidiasis (Biernasiuk et al., 2021). However, other emerging drug-resistant *Candida* species—*non-albicans Candida* spp. like *C. krusei*, *C. tropicalis or C. parapsilosis—* worsen the problem (Pristov and Ghannoum, 2019; Jamiu et al., 2021). The current available antifungal drugs are limited, only including polyenes (e.g., amphotericin B) that bind fungal cell membrane ergosterol, leading to cell lysis, triazoles (e.g., fluconazole, itraconazole) that inhibit ergosterol biosynthesis, and echinocandins (e.g., caspofungin) that inhibit glucan synthesis (Caballero et al., 2021). Nevertheless, most of them, especially fluconazole, collectively suffer from drawbacks such as toxicity and drug resistance (Galdiero et al., 2019; Rybak et al., 2020). Hence, there is an urgent need to develop novel, efficient, and low toxic antifungal agents against pathogenic *Candida* spp.

Antimicrobial peptides (AMPs) have been regarded as potential sources for the discovery of novel drugs, owing to their properties of innate immune components in invertebrates, vertebrates, and plants (Wang et al., 2015; Sowa-Jasiłek et al., 2020). Recently, AMPs have attracted great interests due to their broad-spectrum antimicrobial activities, low toxicity, small molecules, and low weight cationic peptides (Bosch and Zasloff, 2021). To date, many AMPs have been isolated and characterized for their antifungal activity (Lázár et al., 2018; Aguiar et al., 2020; Cheng et al., 2020). Today, more than 3,000 AMPs have been found in different AMP databases, while only a few peptide-based drugs are available in the market and/or preclinical stage (Seyedjavadi et al., 2019). Revealing the mechanism of action of AMPs motivate the discovery and commercializing novel potent AMPs as generation therapeutic drugs (Mahlapuu et al., 2016).

The mechanisms of antifungal drugs are mainly associated with disruption of fungal cell wall, damage of cell membrane, or disturbance of intracellular components (Norris et al., 2021; Orrapin et al., 2021; Seyedjavadi et al., 2021). The action of AMPs was closely related to their special structures. Most of the AMPs adopt an amphipathic α-helical structure and have a membrane mimetic mechanism of action. For example, P19, a central-symmetric α-helix structural peptide, displayed disruptive effects on fungal cell membrane physiology by competitive interaction with the plasma membrane (Chou et al., 2021). Another Dual-Targeted α-helical peptide exerted antifungal effects due to membrane disruption and induction of reactive oxygen species (ROS) production (Yang et al., 2020). Causally, AMPs exert antifungal activity through interactions with target cell membranes, leading to membrane depolarization, membrane permeabilization, or leakage of intracellular components, eventually causing cell death (Lv et al., 2014; Seyedjavadi et al., 2019). It has been known that ROS generation is usually along the process of cell death when microorganisms are threatened by adverse factors, such as environmental stimuli or drug stress, while ROS production is associated with mitochondrial function. Mitochondria supply energy to the cell for metabolism by producing adenosine triphosphate (ATP), which also plays an important role in oxygen consumption and Ca^2 +^ mobilization. However, when mitochondria are damaged by ROS, causing oxidative stress that induces cell apoptosis or death, more ROS are produced in the cells (Zorov et al., 2014). Meanwhile, the mitochondria is also a dynamic Ca^2 +^ store to regulate the balance of the mitochondria and cytoplasm (Juvvadi et al., 2017). It has been reported that AMPs disorder mitochondrial homeostasis, including mitochondrial membrane potential (△Ψ_m_) loss, ATP depletion, or abnormal Ca^2 +^ transition, leading to cell apoptosis (Tian et al., 2017; Saibabu et al., 2020). The antifungal mechanisms of target on mitochondria provide a valuable strategy for developing antifungal drugs.

Cecropins are small helical secreted peptides with antimicrobial activity that are widely distributed among insects (Carboni et al., 2021). Previously, we reported a new member of the cecropin family, the Cecropin 4 (Cec4), which has a significant bactericidal effect on clinical carbapenem-resistant *A. baumnnii* and can eradicate its mature biofilm (Liu et al., 2020). However, as a narrow antibacterial spectrum and a long 41 amino acid chain, the commercial application of Cec4 remains challenging. In the previous study, Cec4 was truncated and modified by varying the charge and hydrophobicity balance. Among the Cec4-derivants, C18, which maintained antibacterial structure of α-helical, only owned 16 amino acids, but had a broader antibacterial spectrum than the parent peptide. Interestingly, C18 not only exhibited a potent antibacterial efficacy on Methicillin-Resistant *Staphylococcus aureus* (MRSA) by altering the membrane potential, increasing fluidity and membrane breakage, but also showed significant antifungal effects on *C. albicans* reference (Peng et al., 2021). Nevertheless, whether C18 has the same antifungal efficacy against other clinical *Candida* isolates, including drug-resistant strains, and the mode of C18 on *C. albicans* remained unclear.

Therefore, following assays were carried out: drug susceptibility tests were examined to evaluate the effect of C18 on *Candida* species; experiments of cell membrane integrity, ROS generation, and mitochondrial function were performed to elucidate the underlying antifungal mechanisms of C18 against *C. albicans*; and *Galleria mellonella* (*G. mellonella*) model was used to assess the therapeutic efficacy of C18 *in vivo*. Finally, we confirmed that C18 triggered mitochondrial dysfunction induced by excessive ROS generation which was driven by plasma membrane damage and attenuated potential virulence traits. Moreover, low toxicity *in vivo* and enhanced survival of *C. albicans*-infected *G. mellonella* model further validates its *in viv*o efficacy. Keeping in view the pleiotropic effects displayed by C18 treated cells with dysfunctional mitochondria, we considered that targeting mitochondria would have therapeutic implications in *C. albicans*.

## Materials and Methods

### Peptide and Reagents

The novel synthesized Ceropin-4 derived peptide, C18(LWKIGKKIWRVLWNWR), showed high activity against *C. albicans*, with reference in our previous study (Peng et al., 2021). Consequently, C18 was synthesized by Gil Biochemical Co., Ltd. (Shanghai, China) with purity >95%, as verified by high-performance liquid chromatography (HPLC) and mass spectrometry. Fluconazole (FLZ) was purchased from Sigma-Aldrich. Other reagents were obtained from solarbio (Beijing, China) unless indicated otherwise.

### Fungal Strains and Growth Conditions

The yeast-like fungus *C. albicans* (*C. albicans* reference strain SC5314 and clinical isolates from the human blood) were preserved by the Key Laboratory of Modern Pathogenic Biology of Guizhou Medical University. Nine clinical isolates from patients’ blood with invasive infection were from the Clinical Laboratory Center in the Affiliated Hospital of Guizhou Medical University, including *C. albicans* (2 strains), *C. krusei* (2 strains), *C. tropicalis* (2 strains), and FLZ-resistant *C. tropicalis* (3 strains), respectively. In addition, the strain ATCC 22019 (*Candida Parapsilosis*) was used for monitoring susceptibility. All strains were grown in a medium of 1% yeast extract, 2% peptone, 2% dextrose (YPD medium) at 30°C and stored on YPD plates solidified with 1.5% agar at 4°C. A single *C. albicans* colony was inoculated into YPD broth and incubated at 35°C overnight with 200 rpm of shaking and grown to the exponential phase for further experiments. For hypha growth of *C. albicans*, RPMI-1640 (Invitrogen, Carlslad, CA, United States) supplemented with 15% fetal bovine serum (FBS) (Sigma-Aldrich) was used as culture medium.

### Determination of Minimal Inhibitory Concentration

Minimum inhibitory concentration (MIC) for C18 against the *Candida* strains was tested by broth micro-dilution assay in a 96-well microtiter, according to the procedures of the Clinical and Laboratory Standards Institute (CLSI) standard (Testing and Guideline, 2008), with slight modifications. For one thing, the exponential fungal cells incubated in YPD medium overnight were washed twice by sterile phosphate buffer saline (PBS; pH7.4), and then diluted to 1∼2 × 10^3^ CFU/ml using a blood cell counting plates. For another, the tested concentrations of C18 were range from 0.25 to 128 μg/ml. After which, *Candida* cells were incubated with C18 in Sabouraud-dextrose broth (SDB) and fungal growth or inhibition was observed after 24 h. To be specific, 200 μl of cell suspension containing the certain concentration of C18 were added to the 96-well microplate and incubated at 35°C for 24 h. Broth with FLZ and un-inoculated broth were used as positive control and negative control, respectively. Finally, the minimal inhibitory concentration (MIC) was determined as the lowest concentration at which no visual growth was observed, corresponding to 90% inhibition of fungal growth. The experiments were repeated in triplicate and three multiple wells were set up in each experiment.

### Growth Inhibition Kinetics and Killing Kinetics

To analyze antifungal or fungicidal process of C18 against *C. albicans*, the time-kill kinetics of the peptide on *C. albicans* SC514 was further investigated by observing the cells survival at different times after C18 treatment. Briefly, *C. albicans* suspension on logarithmic phase was diluted to 2 × 10^6^ cells/ml and incubated with C18 at concentrations (1∼4 × MIC) at 35°C for 48 h. The controls were consistent with the MIC assays on the above. During the cocultivation period, OD_630_ was recorded by a microtiter Absorbance Reader at 2 h interval. Meanwhile, the culture was removed at specific time intervals (0 h, 1 h, 2 h, 3 h, 4 h, 5 h, 6 h, 7 h, and 8 h), diluted serially by 10-fold using PBS, and spotted on solid YPD plate using 10 μl diluted suspension. Fugal colonies were counted after incubation at 35°C for 24 h. The results were presented as the average of triplicate measurements from three independent assays. For the results analysis, colonies of the culture at the corresponding cultivation time was used to express the rate and extent of killing fungi.

### *In vitro* Antibiofilm Assay

The antibiofilm effect of C18 was measured through a 2,3-bis(2-methoxy-4-nitro-5-sulfophenyl) 2H-tetrazolium-5-carboxanilide sodium salt (XTT) reduction assay as previous described with slight modification (Liu et al., 2017). Briefly, 100 μl of *C. albicans* cells solution at the density of 2 × 10^6^ CFU/ml was incubated in RPMI-1640 medium in a 96-well flat-bottomed polypropylene plate at 37°C for 90 min for initial adhesion. Nonadherent cells were removed and fresh RPMI-1640 medium with or without different concentrations of C18 was added, followed by another 24 h of incubation. For examination of C18 on preformed biofilms, biofilms pre-grown for 24 h were exposed to different concentrations of C18 and incubated for another 24 h at 37°C. After incubation, the supernatant was discarded and 100 μl of newly prepared XTT (Shanghai yuanye, China) solution was added to each wall and incubated for 2 h in the dark at 37°C. Next, the colored supernatant from each well were transferred to a new 96-well flat bottom plate and detected by a microplate reader at 490 nm for determination of the antibiofilm effect of C18. The assay was performed in triplicate. Finally, the percent of biofilm viability normalized to the no-treatment control was calculated as follows:


$$\left(\%\right)\text{Biofilm}\text{Viability}=\left(\frac{{\text{A}}_{490}^{\text{treated}}-{\text{A}}_{490}^{\text{medium}}}{{\text{A}}_{490}^{\text{untreated}}-{\text{A}}_{490}^{\text{medium}}}\right)\times 100$$

where $A_{490}^{t\,r\,e\,a\,t\,e\,d}$ is the mean absorbance for wells containing C18 or FLZ, $A_{490}^{u\,n\,t\,r\,e\,a\,t\,e\,d}$ is the mean absorbance of untreated cells, and ${\text{A}}_{490}^{\text{medium}}$ is the mean absorbance of wells containing medium.

### Morphogenetic Observation

To analyze the effect of the peptide on the transition of yeast-to-hyphal in *C. albicans*, inverted microscope assay was carried out according to the previous described protocol (Wu et al., 2020). Exponentially growing *C. albians* were harvested and diluted to 2.0 × 10^6^ CFU/ml in RPMI-1640 medium. In order to hyphal growth, 15% Fetal Bovine Serum was added to the medium. Then, C18 at the concentration of 32 μg/ml and 64 μg/ml were added to a 24-well flat and incubated at 37°C for 2 h, 4 h, and 6 h. The differences in microscopic observation studies among groups were observed using a high-power (× 400) and photographed by an OLYMPUS IX51 microscope (Tokyo, Japan). For examining the inhibitory degree of C18 hyphal formation, at least 300 *C. albicans* cells incubated with or without C18 for 6 h were counted to quantify the hyphal formation rate.

### Scanning Electron Microscopy

Scanning electron microscopy (SEM) was applied to evaluate the surface morphological changes of *C. albians* caused by C18 as the previous report (Lyu et al., 2016). Briefly, 2.0 × 10^6^ CFU/ml of *C. albians* on exponential phase in YPD medium was exposed to 32 μg/ml and 64 μg/ml C18 for 3 h at 35°C. After incubation, the cells were centrifuged for 10 min at 5,000 rpm, washed twice in PBS, and then post-fixed with 2.5% glutaraldehyde at 4°C overnight. Next, the fixed samples were washed with PBS two times, followed by dehydration using tertiary butyl alcohol series (50%, 75%, 95%, and 100%) for 10 min. Subsequently, the samples were dried in the high vacuum evaporator and coated with a thin layer of gold-palladium. Finally, the prepared sample was placed in to Hitachi H-7650 Scanning Electron Microscope (Tokyo, Japan) for observation (×2,000 and ×5,000) to obtain images.

### Measurement of Cell Membrane Permeability

Fluorescent Intensity and Confocal Laser Scanning Microscopy (CLSM) Assays were used to quantify and visualized the membrane permeability in *C. albicans*, respectively. The propidium iodide (PI), a non-vital nuclear stain used for identifying dead cell, was used to analyze the membrane integrity as previously described (Seyedjavadi et al., 2019). Briefly, 2.0 × 10^6^ CFU/ml of *C. albicans* on logarithmic growth in YPD broth was exposed to 32 μg/ml and 64 μg/ml C18 for 3 h, 6 h, and 12 h at 35°C, shaking at 120 rpm. Next, 10 mM Propidium iodide (PI) (Sigma, United States) solution was added, and further incubation was done for 15 min at 35°C in the dark, followed by PBS washing. Finally, the membrane permeability assay was assessed by fluorescence intensity of PI (excitation = 525 nm, emission = 590 nm) that was measured by RF-5301PC sectrofluoro-photometer (Bio-Tek SynergyHTX, United States).

For direct visualization of *C. albicans* membrane, PI and SYTO9 (Invitrogen, United States) were also used to distinguish apoptotic or living cells, and the specimens treated with C18 or not were examined using Olypus CLSM (Markham, ON, Canada). As opposed to PI, SYTO9 is a fluorescent molecule that produces green fluorescence at the excited wavelength of 488 nm, indicating live cells. In brief, the log-phase *C. albicans* (2.0 × 10^6^ CFU/ml) in YPD medium was exposed to 32 μg/ml and 64 μg/ml C18 for 6 h at 35°C, shaking at 120 rpm. Afterward, the suspension was co-incubated with PI (10 μg/ml) and SYTO9 (10mM) for 15 min at 37°C in the dark, followed by PBS washing. Next, a volume of 5 μl of suspension was added on a sterile polylysine-treated glass slide, followed by circular cell cover glass that was covered immediately. Finally, the images were taken with an Olympus CLSM (Markham, ON, Canada) at an excitation wavelength of 488 nm for SYTO9 and 525 nm for PI.

### Intracellular Reactive Oxygen Species Production

In order to analyze the efficacy on the redox of *C. albicans* treated with C18, ROS was examined using 2′,7′-dichlorofluorescein diacetate (H_2_DCFDA) as previously described (Dias et al., 2020). Briefly, 2.0 × 10^6^ CFU/ml of *C. albians* on exponential phrase in YPD broth was exposed to 32 μg/ml and 64 μg/ml C18 for 3 h, 6 h, and 12 h at 35°C. After incubation, the fungal cells were washed using sterile PBS, adjusted to 2.0 × 10^6^ CFU/ml, and then stained with 10 μM H_2_DCFDA for 30 min at 37°C in dark. Next, the samples were washed twice using PBS, followed by suspending the cells. Finally, the suspension was detected by RF-5301PC sectrofluoro-photometer (excitation = 490 nm, emission = 530 nm). In addition, the samples exposed to C18 for 6 h were collected to be observed for CLSM.

### Cytosolic Ca^2 +^ Assays

To evaluate the defect in intracellular *Ca*^2 +^ homeostasis of *C. albicans* induced by C18, Fluo-3/AM was used to measured cytosolic Ca^2 +^ levels as previously described (Chen L. et al., 2019). Briefly, 2.0 × 10^6^ CFU/ml of *C. albians* on logarithmic phrase in YPD broth was exposed to 32 μg/ml and 64 μg/ml C18 for 3 h, 6 h, and 12 h at 35°C. After harvest, the cells were washed twice in sterile PBS and resuspended in 500 μl of Hanks balanced salt buffer solution (HBSS). Next, 2 μM of Fluo-3/AM was added to the suspension, then co-incubated for 40 min at 30°C in darkness. Afterward, the mixture was washed once, resuspended in 600 μl HBSS, and incubated at 30°C for further 20 min. Finally, the fluorescence intensities of Fluo-3 AM were examined by RF-5301PC sectrofluoro-photometer (excitation = 490 nm, emission = 530 nm). In addition, the culture exposed to C18 for 6 h was prepared to be observed by CLSM.

### Mitochondrial Membrane Potential (△Ψm) Assays

The changes in the mitochondrial membrane potential (MMP, △Ψ_m_) of *C. albicans* treated with C18 was measured using 5,5′,6′,6′-terachloro-1,1′3,3′-tetraethyl-benzimidazolyl carbocy-anine iodide (JC-1) (Beyotime, China) as previously described (Chang et al., 2021). In healthy cells, the lipophilic and positively charged JC-1 dye as JC-aggregate accumulates in the negatively charged mitochondria, which leads to red fluorescence, and green fluorescence of the JC-monomer inside the cytosol if otherwise. When the △Ψ_m_ of *C. albicans* was lost or depolarized, the cell apoptosis prevents JC-1 accumulation in the mitochondria, and the apoptotic cells only presents green fluorescence. In brief, 2.0 × 10^6^ CFU/ml of *C. albians* on logarithmic phrase in YPD broth was exposed to 32 μg/ml and 64 μg/ml C18 for 3 h, 6 h, and 12 h at 35°C. Carbonyl cyanide m-chlorophenyl hydrazine (CCCP), a mitochondrial uncoupler, can depolarize △Ψm of cells and was used as the positive control. After harvest, the cells were washed twice in sterile PBS and stained with 2.5 μg/ml JC-1 for 40 min in the dark at 37°C. Next, the mixture was washed and measured by RF-5301PC sectrofluoro-photometer. Notably, the ratio of the fluorescence intensities of JC-aggregate (excitation = 525 nm, emission = 590 nm) to JC-monomer (excitation = 490 nm, emission = 530 nm) was calculated to confirm whether C18 induced the △Ψ_m_ of *C. albicans* loss. In addition, the culture exposed to C18 for 6 h was prepared to be observed by CLSM.

### Detection of Intracellular Adenosine Triphosphate Levels

In order to understand the mitochondrial energetic metabolism of *C. albicans* treated with C18, intracellular ATP levels was measured using ATP assay kits (Beyotime China) according to the manufacturer’s instructions (Chen Y. W. et al., 2019). Briefly, 2.0 × 10^6^ CFU/ml of *C. albians* on logarithmic phrase in YPD broth was exposed to 32 μg/ml and 64 μg/ml C18 for 3 h, 6 h, and 12 h at 35°C. After harvest, the cells were washed twice in sterile PBS and adjusted to 2 × 10^6^ CFU/ml in YPD medium. Subsequently, ATP levels were calculated with reference to the standard curve, and the results were expressed as nmol/mg protein.

### *In vivo* Evaluation of Therapeutic Activity of C18 Against *Candida albicans*

*In vivo* potential therapeutic effects of C18 were assayed in *G. mellonella* larvae and subjected to a lethal dose of *C. albicans* SC5314 cells, as to previously described (Fernandes et al., 2020). *G. mellonella* larvae at their final instar stage were purchased from Tianjin Huiyude Biological Technology Co., Ltd., each weighing 250∼300 mg with length of approximate 2∼3 cm. All larvae were placed in the dark incubator at 35°C overnight before the experiment. For the assessment of the peptide toxicity, group of 10 larvae at their final instar stage were injected (10 μl per larva) with C18 at the various concentrations range from 4 to 32 mg/kg. The same volume of sterile PBS and FLZ (4 mg/kg) was used as negative control and positive control. For the evaluation of peptide therapeutic activity, 5 × 10^7^ CFU/ml of *C. albicans* SC5314 on logarithmic phrase in sterile PBS were prepared, and 10 μl of *C. albicans* suspension was injected into each larva through its last left proleg. One hour later, 10 μl of tested drug were injected into its last right proleg. Then, the larvae were incubated at 35°C for 5 days, with live and dead counts being performed every 24 h. We considered that they were dead when larvae became black or soft and there was no obvious tactile response. Survival curves of peptide-treated and control larva were analyzed by the Mantel-Cox log-rank test. A value of *p <* 0.05 was considered significant. Meanwhile, fungal burden of *G. mellonella* larvae was determined. After C18 injection for 24 h, 3 larvae were selected randomly in each group (regardless of whether they were dead or alive), placed in 3 ml sterile PBS solution, and grinded with a high-speed homogenizer. The homogenate was diluted for serial 10 times, and 10 μl of homogenates of various concentration gradients were spotted on sterile solid YPD medium. The number of fungal cells were not recorded until the colonies grew on the culture medium (Chen et al., 2021). Then, we converted it to get the *C. albicans* load (CFU per larva) of each larva before dilution.

### Statistical Analysis

Every experiment was independently performed at least three times, and the data were expressed as mean ± SD of three independent experiments. Differences between experimental groups were assessed for significance using One-Way ANOVA with GraphPad Prism 8 software. For the *G. mellonella* survival experiment, Long-rank was used in the analysis of Mantel-Cox survival curves. The *p<0.05,^**^p<0.01, and ^***^p<0.001 levels were considered to indicate statistical significance.

## Results

### Characterization of C18

**FIGURE 1:**
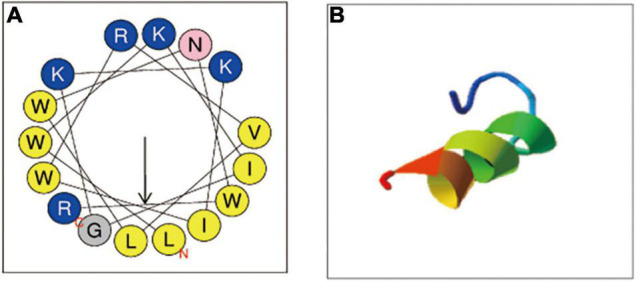
Physicochemical properties of C18. **(A)** The helical wheels of C18. The helical wheels were constructed with NetWheels (https://heliquest.ipmc.cnrs.fr/cgi-bin/ComputParams.py). **(B)** The predicted 3D conformation of the synthetic peptide C18 (https://zhanggroup.org/I-TASSER/).

C18, derived from cecropin 4 (Cec4) with 41 amino acids, maintained the structure of the N-terminal helix of most cecropins starts from the Lys residue (Yagi-Utsumi et al., 2013), and owned 16 amino acid (Figure 1A). For the properties of C18, mass spectrometry analysis confirmed its molecular weight 2181.73 Da and purity of 95.53% (Supplementary Figure 1). In addition, we analyzed the physical and chemical parameters of C18 through the ExPASy Bioformatics Resource Portal^1^, and obtained its 3D image of secondary structure predicted using ITASSER application (Figure 1B)^2^. Its net charge at physiological pH and Hydrophobicity was + 5 and 0.73, respectively (Table 1).

**TABLE 1:** Amino acid sequence and physicochemical properties of C18.

Peptide	Amino acid sequence	Purity	Molecular Mass (Da)	Net charge (physiological pH)	Hydrophobicity
C18	LWKIGKKIWRVLWNWR	95.53%	2181.73	+ 5	0.73

### Antifungal Activity of C18

Although *C. albicans* remains the main cause of candidiasis, in recent years, a large number of infections has been attributed to non-albicans *Candida* species, including *Candida krusei*, *Candida tropicalis*, etc. (Barac et al., 2020; Jamiu et al., 2021). Furthermore, the emergence of drug-resistant *Candidas* was closely related to the high mortality of fungi (Gold et al., 2021). To further verify the efficacy of C18, the susceptibility of the peptide on ten *C. albicans* and non-*albicans Candida* clinical isolates was tested by the broth dilution method. Additionally, we adopted FLZ as the positive control to compare the antifungal susceptibility. As shown in Table 2, C18 exhibited significant antifungal activity against *C. albicans, Candida krusei, Candida tropicalis*, and *Candida parapsilosis*, with MIC values ranging from 4 to 32 μg/ml, and the MIC of C18 on the clinical isolates was similar to or lower than that *C. albicans* SC5314 reference. Amazingly, C18 had more powerful antifungal effect to clinical isolates of *C. tropicalis*, especially to the FLZ-resistant *C. tropicalis* isolates with an MIC value of 8 or 16 μg/ml. These results demonstrated that C18 exerted well antifungal activity on both *C. albicans* and non-*albicans Candida* species, even FLZ-resistant ones.

**TABLE 2:** Minimal growth inhibitions (MICs) of C18 against fungus.

Microbial strains	MIC (μg/ml,μM)	
	
	C18	FLZ
*Candida albicans* SC5314	32/14.67	4/1.83
*Candida albicans* ATCC 10231	32/14.67	4/1.83
*Candida albicans* 16102	16/7.33	4/1.83
*Candida albicans* 16229	16/7.33	4/1.83
*Candida krusei* 7	32/14.67	32/14.67
*Candida krusei* 17	32/14.67	16/7.33
*Candida tropicalis* 11	16/7.33	64/29.33
*Candida tropicalis* 78	8/3.66	64/29.33
FLZ-resistant *Candida tropicalis* 8402	16/7.33	>128/58.67
FLZ-resistant *Candida tropicalis* 6984	16/7.33	>128/58.67
FLZ-resistant *Candida tropicalis* 4252	8/3.66	>128/58.67
*Candida parapsilosis*	4/1.83	4/1.83

### Growth Inhibition and Killing Kinetics

**FIGURE 2:**
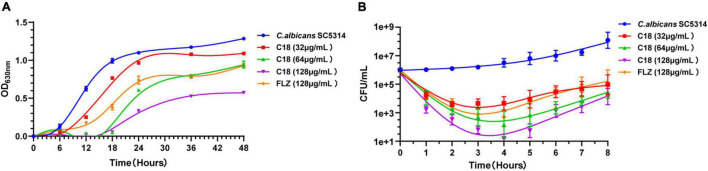
Inhibition and killing kinetics of C18 against *Candida albicans*. **(A)** Growth curve of *C. albicans* within incubation for 48 h. Optical density at 630 nm (OD_630nm_) of *C. albicans* SC5314 in the presence or absence of C18 at the concentration of 32∼128μg/ml was measured. **(B)** Time-killing kinetics of C18 against *C. albicans* SC5314. Fungal cells were incubated with C18 at concentrations of 32∼128 μg/ml for 8 h, and the colonies of *C. albicans* suspension were counted at every 2 h. The drug-free strain-containing medium was set as the control and fluconazole (FLZ) was used as the positive drug. Each data point represents mean ± SD of three independent experiments.

Antimicrobial peptides seem to be capable of fast killing fungal cells (Ciociola et al., 2021). From the growth curve of *C. albicans* (Figure 2A), in the absence of C18, *C. albicans* entered the logarithmic phase within 6 h of incubation and reached stationary phase at 18 h. But the logarithmic phase of *C. albicans* treated with C18 just only started after 18 h of incubation at the concentration of 2MIC and 4MIC. C18 showed significant concentration-dependent inhibition of *C. albicans*. In order to clearly observe the anticandidal efficacy of C18 in the early phase, the colonies of *C. albicans* was counted in the first 8 h of incubation (Figure 2B). Similarly, C18 showed excellent fungistatic effects against *C. albicans* during the first 4 h when exposed in the concentration of 1∼4 MIC compared with the control. Moreover, the colonies of *C. albicans* reduces more than 10^4^ CFU/ml when treated with 4MIC of C18 after 4 h of incubation. The kinetics of growth and killing curve indicated that C18 can retard *C. albicans* reaching to logarithmic phrase and have a fast antifungal effects on *C. albicans* in the early stage.

### C18 Inhibits Biofilms of *Candida albicans*

**FIGURE 3:**
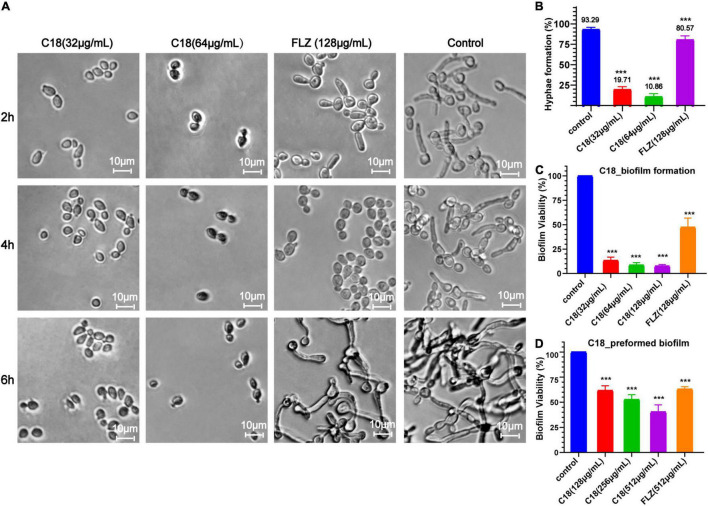
Effect of C18 on morphogenesis and biofilm in *Candida albicans*. **(A)** The hyphal formation of *C. albicans* exposed to C18 was detected by invert microscope. To induce hyphae formation, RPMI-1640 with 15% FBS was added and incubated at 35°C for 2 h, 4 h, and 6 h. C18 was treated at 32 μg/ml and 64 μg/ml, the drug-free strain-containing medium was set as the control, and 128 μg/ml FLZ was set as the positive control. **(B)** The morphogenesis inhibitory effect of C18 was evaluated and recorded as a percentage. *Candida albicans* cells was incubated with C18 (32 μg/ml and 64 μg/ml) or FLZ (128 μg/ml) in RPMI-1640 medium containing 15% FBS at 35°C for 6 h, and at least 300 cells was counted using a microscope. *C. albicans* cells without any treatment were set as control. **(C,D)** Effect of C18 on biofilm formation **(C)** and preformed biofilm **(D)** in *C. albicans* by the 2,3-bis(2-methoxy-4-nitro-5-sulfophenyl) 2H-tetrazolium-5-carboxanilide sodium salt (XTT) reduction assay. For biofilm formation, *C. albicans* cells was incubated with C18 (32 μg/ml, 64 μg/ml, and 128 μg/ml) or FLZ (128 μg/ml) in RPMI-1640 medium at 37°C for 24 h. For preformed biofilm, *C. albicans* cells was incubated in RPMI-1640 medium at 37°C for 24 h to form mature biofilm. Next, supernatant was discarded and C18 (128 μg/ml, 256 μg/ml, and 512 μg/ml) or FLZ (512 μg/ml) was added and co-incubated for another 24 h. XTT reduction was performed to determine the level of biofilm viability at various treatment and colorimetric absorbance was measured at 490 nm. Error bars represent standard deviation of three independent experiments. ***p < 0.001 compared to the control group.

Both morphogenesis and biofilm formation are the important virulence of *C. albicans*, which are closely related to pathogenicity. Hyphal elements are the main structures embedded in mature *C. albicans* biofilm (Vavala et al., 2013). The morphological changes of *C. albicans* hyphae are illustrated in Figure 3A. In the control group, the length and density of *C. albicans* hyphae increased along with the incubation time, and the hyphae of *C. albicans* cells grew so long to link across and connect forming a network after incubation for 6 h. However, when treated with 32 μg/ml or 64 μg/ml of C18, large-scale hyphae of *C. albicans* were completely inhibited, and only single yeast-like cells were observed. Nevertheless, FLZ only had slight inhibition activity on the hyphal structure during the observed period. Meanwhile, we quantified the percentage of hyphae formation of *C. albicans* cells in each group after treatment for 6 h (Figure 3B). Hyphae were formed in 93.29% of cells in the control group, while hyphae were formed in only 19.71% and 10.86% of cells when exposed to 32 μg/ml and 64 μg/ml of C18, respectively.

The ability of *Candida* to form biofilms is implicated in its pathogenicity and resilience to treatments because cells within the biofilm are physically protect from host immune responses and antifungal drugs (De Zoysa et al., 2018). We employed XTT reduction assay to evaluate the antibiofilm effect of C18. Bar diagram showed that C18 significantly reduced the percent of biofilm viability in a concentration-dependent manner. Compared to the control group, C18 at the concentration of 32 μg/ml, 64 μg/ml, and 128μg/ml inhibited biofilm formation by 87%, 91%, and 92%, respectively (Figure 3C). Nevertheless, FLZ only inhibited about 50% biofilm of *C. albicans*. The great antibiofilm activity of C18 necessitated further integration the eradication effect of C18 on preformed biofilm. When the preformed biofilm (24h) was exposed to C18, we found 128 μg/ml, 256 μg/ml, and 512μg/ml of C18 reduced mature biofilms by 39%, 48%, and 57%, respectively (Figure 3D). Therefore, the antibiofilm effect of C18 was confirmed that 80% of Minimum Biofilm Inhibitory Concentration (MBIC_80_) was 32 μg/ml and 50% Minimum Biofilm Eradication Concentration (MBEC_50_) was 512 μg/ml. These results suggested that C18 can significantly inhibit biofilm formation in *C. albicans* attributed to its inhibitory effect of yeast-to-hyphae transition and can eliminate a certain amount of mature biofilm.

### C18 Effectively Treat *Candida albicans* Infection *in vivo*

**FIGURE 4:**
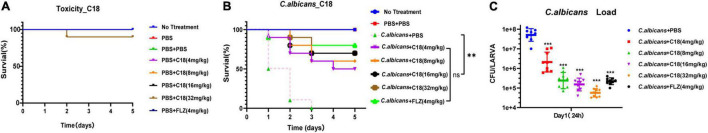
*In vivo* toxicity and therapeutic activity of C18 in a *Galleria mellonella* model. **(A)** The toxicity of C18 in *G. mellonella* larvae model. A range of concentrations (4 mg/kg∼32 mg/kg) of C18 injected to the larvae (10 μl pre larva) was applied to evaluate toxicity of the peptide. Only 10% of the larvae died when treated with 32 mg/kg C18 for 5 days. **(B)** Survival assays for 3 replicate experiments combined (*n* = 30). *Galleria mellonella* larvae were infected with 10 μl inoculum of 5 × 10^7^ CFU/ml of *Candida albicans* and treated with various concentrations of C18 or 4 mg/kg FLZ. For each replicate, a group of 10 larvae in each treatment was monitored for survival over 5 days. **p < 0.01, compared to the group of *C. albicans* + phosphate buffer solution (PBS). **(C)** Fungal burden for three replicate experiments (*n* = 3). For each replicate, three larvae from each group were sacrificed at 24 h post treatment, homogenized, cultivated on yeast extract peptone dextrose (YPD) medium. Colonies are counted and converted to obtain to the *C. albicans* counts per larva. ***p < 0.001 compared to the group of *C. albicans* + PBS.

Toxicity to the host and therapeutic activity against *C. albicans* infection were evaluated for C18 in *G. mellonella* larvae. First, toxicity was determined using a series of increasing doses (4, 8, 16, and 32 mg/kg). As shown in Figure 4A, all the larvae were alive at 5 days, except that 10% of larvae injected with 32 mg/kg of C18 died on the second day. However, there is no significant difference in the survival between the larvae injected with PBS (the control group) and the various dose of C18, suggesting that the peptide was not toxic in this experimental model. Next, the therapeutic effect of C18 was evaluated in larvae with a lethal inoculum of *C. albicans* (5 × 10^7^ CFU per larva), and the survival percentage and *C. albicans* burdens were measured. After *G. mellonella* larvae were infected with *C. albicans*, the above concentration of C18 was applied to treat the infected larvae and improve the survival significantly (*p<*0.01) (Figure 4B). Interestingly, the groups of 16 mg/kg and 32 mg/kg both had 70% survival at 5 days post infection, indicating that even higher than 16 mg/kg of C18 treatment cannot produce a super survival of *C. albicans*-infected larvae, which provides us some insight on the boundary of efficacy and toxicity. Correspondingly, *C. albicans* burden mirrored these results (Figure 4C), the number of colonies pre larvae treated with C18 for 24 h decreased significantly in all the agent-treatment groups (*p<*0.001). Moreover, 16 mg/kg of C18 reduced fungal burden more than 10^2^ CFU pre larva than the group without treatment, similar to 4 mg/kg of FLZ treatment. Notably, even though 32 mg/kg of C18 had the same improved survival with 16 mg/kg of C18, the *C. albicans* burden of the group in the dose of 32 mg/kg of C18 was lower than other treated groups. All the results indicated that C18 has no obvious toxicity *in vivo*, and the peptide is significantly efficacious in *C. albicans*-infected *G. mellonella* model.

### C18 Damages Cell Structure of *Candida albicans*

**FIGURE 5:**
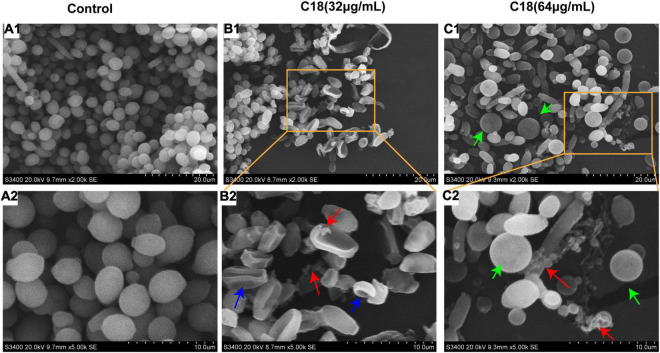
Scanning electron microscopic (SEM) images of *Candida albicans* in the absence and presence of C18 at 35°C for 3 h. **(A1-A2)** SEM images of *C. albicans* treated without C18. **(B1-B2)** SEM images of *C. albicans* treated with 32 μg/ml of C18. **(C1-C2)** SEM images of *C. albicans* treated with 64 μg/ml of C18. Red arrow indicates the cell wall and membrane was destroyed leading to intracellular component leaked away. Blue arrow indicated the surface was folded and depression occurred in the cells. Green arrow indicated the size of cells was different and many cells were swollen.

The cell wall of *C. albicans* consists of an external protein coat and an internal skeletal layer which protects the plasma membrane from damage and acts as a diffusional barrier for many antifungal agents (De Zoysa et al., 2018). SEM was therefore used to investigate whether C18 could destroy the morphological structure of *C. albicans* (Figure 5). It was seen that the cells of the control group were round and smooth, indicating the intact overall structure (Figures 5A1,A2). When the cells were exposed to 32μg/ml or 64μg/ml of C18 for 3h, the cell wall was loosely dispersed, and the surface appeared rough and irregular (Figures 5B1-2,C1-2). In addition, a series of characteristic alterations in *C. albicans*, such as content leakage (**red arrows**), surface folding or depression (**blue arrows**), and different size and cellular swelling (**green arrows**), appeared after C18 treatment. According to the data, it was seemed that C18 could change the morphology of *C. albicans* and damage the structure of cell wall, which was likely to cause intracellular components imbalance.

**FIGURE 6:**
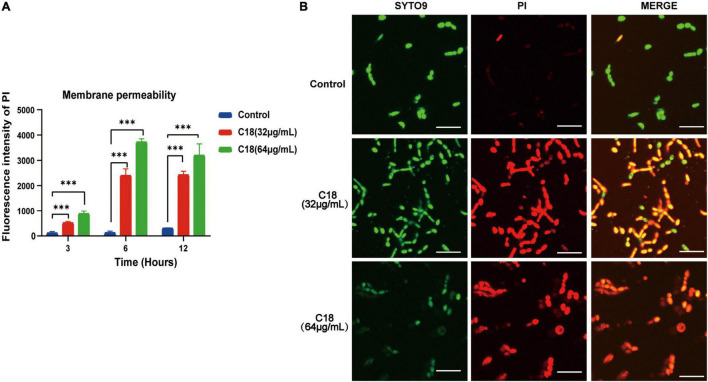
Effect of C18 on the cell membrane permeability of *Candida albicans*. **(A)** Fluorescence intensity of propidium iodide (PI) (10 μg/ml) after C18 treatment was detected by sectrofluoro-photometer. Error bars represent standard deviation of three independent experiments. ^***^p < 0.001 compared to the control group. **(B)** Fluorescence images of *C. albicans* cells stained by SYTO9 (10 μM) and PI (10 μg/ml) after C18 treatment for 6 h using confocal laser scanning microscopy (CLSM). Green represents live microbes while red represents dead microbes, and the merge shown in both green and red are combined from the two channel images. Scale bar 20μm.

Destruction of the cell wall in *C. albicans* observed in the presence of C18 necessitated studying the membrane damage more closely. In this study, we employed PI uptake to investigate the effect of C18 on the fungal cell membrane integrity. PI is a membrane impermeable dye that can only go through damaged membrane of dead cells, binds to the DNA, and produces red fluorescence under excitation light (λ = 532nm), detected by sectrofluoro-photometer. As shown in Figure 6A, the membrane permeability of *C. albicans* cell increased in a dose-dependent manner when exposed to 32 μg/ml or 64 μg/ml of C18 for 3 h, 6 h, and 12 h. In addition, the membrane permeability reached to the top peak after C18 treatment for 6 h. Subsequently, the disturbing effect of C18 on the membrane integrity of *C. albicans* was analyzed by CLSM (Figure 6A). In order to visualize the membrane damage of *C. albicans* cells after C18 treatment, double fluorescent staining of the cells with PI and SYTO9 were used to differentiate dead or live cells. This is because SYTO9 passively crosses through cell membrane, is subsequently hydrolyzed to fluorescein by intracellular esterases, and shows green fluorescence (Saibabu et al., 2020). In the control groups, numerous green cells were visualized, indicating most of the cells had intact membrane integrity. Whereas, the number of viable cells was reduced and the ratio of dead cells stained red to live cells increased dramatically after C18 treatment. Additionally, the membrane damage of *C. albicans* cells exposed to 64 μg/ml of C18 was more severe than 32 μg/ml of C18. The results suggested that C18 facilitated *C. albicans* cells were dead by alteration of membrane permeability.

### C18 Increases Intracellular Reactive Oxygen Species Generation and Cytosolic Ca^2 +^ of *Candida albicans*

**FIGURE 7:**
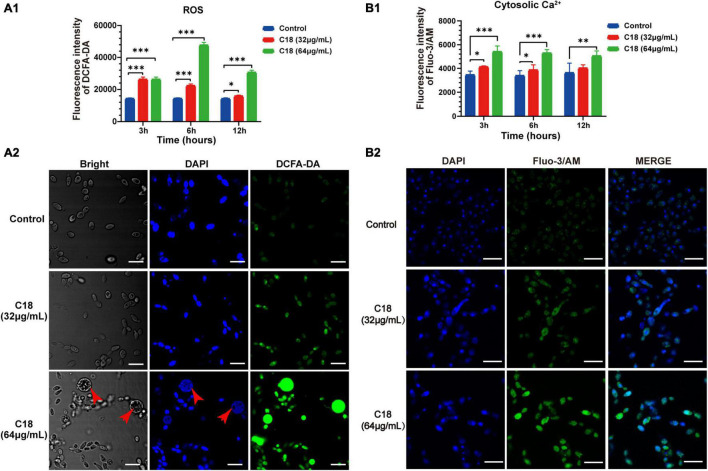
Effect of C18 on intracellular ROS generation and Ca^2 +^ accumulation of *Candida albicans*. **(A1,B1)** Bar diagram showed the level of intracellular ROS and Ca^2 +^ in *C. albicans* treated with C18 at concentrations of 32 μg/ml and 64 μg/ml. The fluorescence intensity of dichlorofluorescein (DCF; indicates ROS) or Fluo-3/AM (indicates cytosolic Ca^2 +^) was detected by sectrofluoro-photometer, respectively. Error bars represent standard deviation of three independent experiments. ^**^p < 0.01,* p < 0.05, ^***^p < 0.001 compared to the control group. **(A2–B2)** Fluorescence images of *C. albicans* cells stained by 2′,7′-dichlorofluorescein diacetate (DCFH-DA) or Fluo-3/AM was captured by CLSM after C18 treatment for 6h respectively. Blue represents cell nucleus stained by 4′,6-diaminidino-2-phenylindole (DAPI), while green represents ROS accumulation **(A2)** and cytosolic Ca^2 +^ **(B2)**, the merge shown in both green and blue are combined from the two channel images. Red arrow indicates dispersive or degradative DNA with DAPI staining in the high level of ROS expression cells. Scale bar 10 μm.

In particular, ROS easily reacts with the membrane lipids and induced lipid peroxidation, leading to increase membrane permeability. In addition, ROS plays as an early initiator of apoptosis in yeast and other filamentous fungi and consequently results in disturbance of cellular oxygen metabolism (Tian et al., 2017; Kim and Lee, 2021). Thus, we proposed that the alteration of membrane permeability induced by C18 (Figure 7) was probably ascribed to intracellular ROS accumulation. For this, 2′,7′-dichlorofluorescein diacetate (DCFHDA), an oxidant sensitive probe, was applied to estimate ROS generation analyzed by sectrofluoro-photometer (Figure 7A1) and CLSM (Figure 7A2). Intracellular ROS is responsible for oxidizing DCFH-DA to dichlorofluorescein (DCF) that produces green fluorescence at the excitation light of 488 nm. Compared to the control group, the ROS production of *C. albicans* cells significantly increased when exposed to 32 μg/ml or 64 μg/ml of C18 for 3 h, 6 h, and 12 h (Figure 7A1). Additionally, the ROS level of *C. albicans* cells after 64 μg/ml C18 treatment for 6 h was nearly three times more than the control group and reached the maximum among the three time-spot of incubation. Meanwhile, such appearance was also seen in CLSM images (Figure 7A2), in which amount of the green cells with ROS-positive staining were seen in C18 treated groups rather than the control group. Notably, when exposed to 64 μg/ml of C18 for 6 h, many cells that appeared much bigger than the cells in other groups exhibited much stronger green fluorescence and produced weak and dispersive blue fluorescence (indicates DNA damage). For ROS accumulation, and thereby oxidative stress, triggers a series of downstream cellular events that lead to disorganization of DNA and therefor cell death (Saibabu et al., 2020). These results indicated that C18 induced intracellular ROS generation, leading to intracellular oxidative damage.

Ca^2 +^ signaling plays an important role in maintenance of redox homeostasis and morphogenesis in the fungal pathogens (Peng et al., 2019). Ca^2 +^ and ROS signaling pathways overlap and impact one another, which both involved in cell stress, damage, or even death (Madreiter-Sokolowski et al., 2020). To investigate the effect of C18 on cytosolic Ca^2 +^ of *C. albicans*, Fluo-3/AM (the cytosolic calcium-specific probe) was used to examine the level of Ca^2 +^ in the cytosol by sectrofluoro-photometer (Figure 7B1) and CLSM (Figure 7B2). Cytosolic Ca^2 +^ can be labeled by Fluo-3/AM that produces green fluorescence at the excitation light of 488 nm. Compared to the control group, the cytosolic Ca^2 +^ of *C. albicans* elevated in a dose-dependent manner when exposed to 32 μg/ml or 64 μg/ml of C18 for 3 h, 6 h, and 12 h (Figure 7B1). Meanwhile, the intensity of green fluorescence labeled cytosolic Ca^2 +^ increased with the increase of the concentration of C18 treatment. Hence, green fluorescence hardly appeared in the control group (Figure 7B2). These results suggested that C18 caused an accumulation of Ca^2 +^ in the cytosol and the disturbance of Ca^2 +^ regulation, which is probably caused by exported movement of Ca^2 +^.

### C18 Disturbs Function of Mitochondria in *Candida albicans*

**FIGURE 8:**
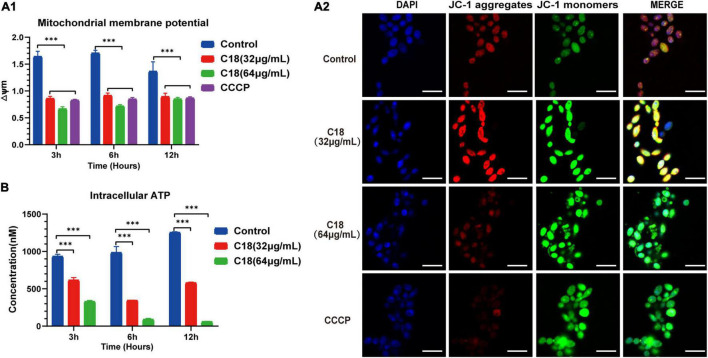
Mitochondrial Dysfunction of *Candida albicans* treated with or without C18. **(A1)** △Ψ_m_ of *C. albicans* was measured by 5,5′,6′,6′-terachloro-1, 1′3,3′-tetraethyl-benzimidazolyl carbocy-anine iodide (JC-1) staining in the presence or absence of 32 μg/ml or 64 μg/ml C18 for 3 h, 6 h, and 12 h. Bar diagram showed the ratio of red fluorescence intensity to green fluorescence intensity (△Ψm) detected by sectrofluoro-photometer. Carbonyl cyanide m-chlorophenyl hydrazine (CCCP), a mitochondrial uncoupler, can depolarize △Ψm of cells and was used as the positive control. The results are presented as mean ± SD of three independent experiments. ^***^p<0.001, compared to the control group. **(A2)** Fluorescence images of *C. albicans* cells stained by DAPI and JC-1 after C18 treatment for 6h was captured by CLSM. Blue represents cell nucleus while red fluorescence represents JC-monomers, and green fluorescence represents JC-aggregates. The color of merged images reflects the degree of △Ψm loss. Scale bar 10 μm. **(B)** Intracellular ATP of cells were measured by sectrofluoro-photometer. The results are presented as mean ± SD of three independent experiments. ^***^p<0.001, compared with the control group.

In general, mitochondria are cellular organelles responsible for generation of chemical energy. In addition, mitochondria are responsible for the cellular adaptation to different types of stressors such as oxidative stress or Ca^2 +^ uptake. When these stressors outstrip the adaptive capacity of mitochondria to restore homeostasis, it causes mitochondrial dysfunction (van der Reest et al., 2021). Mitochondrial Membrane Potential (MMP, △Ψ_m_) represents an essential parameter of cell activities, and is considered as a sensitive marker of the energy-coupling condition of mitochondria (Suski et al., 2018). Reportedly, excessive ROS exposure could encroach on mitochondrial homeostasis and trigger △Ψ_m_ collapse (Mashayekhi et al., 2014). Intracellular ROS accumulation and Ca^2 +^ uptake induced by C18 entails studying mitochondrial dysfunction. First, we employed JC-1 to assess the △Ψ_m_ changes in *C. albicans* cells when C18 treated using sectrofluoro-photometer (Figure 8A1) and CLSM (Figure 8A2). JC-1 is a lipophilic cationic probe with fluorescence that can reversibly change from red (indicates JC-1 aggregates) to green (indicates JC-1 monomers) when the △Ψ_m_ depolarized (Lee and Lee, 2018). As is shown in Figure 8A1, △Ψ_m_ of *C. albicans* cells exposed to 64 μg/ml C18 for 3 h or 6 h reduced two-times less than the control group, similar to the CCCP (a positive agent of △Ψ_m_ collapse)-treated cells. Meanwhile, △Ψ_m_ collapse of *C. albicans* cells in the exposure of C18 was visualized through red and green fluorescence images merged in CLSM (Figure 8A2). These data indicated that C18 promoted △Ψ_m_ depolarization of *C. albicans* and disturbed mitochondrial homeostasis.

Mitochondria supply cells with energy in the form of ATP and dysfunctional mitochondria usually results in depletion of cell ATP pool contributed to cell death (Chistiakov et al., 2018). To investigate the effect on of C18 on the energy metabolism of *C. albicans*, the intracellular contents of ATP were measured using a sectrofluoro-photometer (Figure 8B). Compared to the control group, the APT level in the C18-treated strains markedly decreased in a dose-independent manner. Furthermore, the intracellular ATP content of *C. albicans* cells happened to decrease two-fold less than the control group when exposed to 32 μg/ml of C18 for 3 h and decrease nearly 10 times after 64 μg/ml of C18 treatment for 12 h. △Ψ_m_ depolarization and ATP depletion of *C. albicans* demonstrated that C18 can cause mitochondrial dysfunction under the stress of intracellular ROS generation and Ca^2 +^ accumulation.

## Discussion

The occurrence of invasive fungal infections is significantly increasing, especially in the patients with weak or compromised immune system due to various reasons like organ transplantation or cancer chemotherapy. Seriously, invasive fungal infection results in significant morbidity and mortality, and *Candida* species accounts for approximately half of such infection (Chen et al., 2020). Nevertheless, only a limited number of antifungal agents available in the market make the recent task to deal with *Candida* infection more challengeable. Furthermore, the emergence of multidrug-resistant isolates to conventional antibiotics and their inherent toxicity to humans worsen the problem (Vahedi-Shahandashti and Lass-Flörl, 2020). Therefore, there is an urgent need to discover new classes of effective, safe, well-tolerated broad-spectrum antifungal drugs. Recently, AMPs, as a new kind of potential antifungal candidates, have attracted much attention (Cheng et al., 2020). This study presents the antifungal potential of C18 and its probable mode of actions. Previous studies in our laboratory have found that a Cec4 derived peptide named C18 (16 amino acids) had a broad-spectrum against microorganisms including Gram-positive bacterial, Gram-negative bacteria, and yeast (Peng et al., 2021). However, scientifical validation of its antifungal effects and understanding the mode of action on a prevalent human fungal pathogen like *C. albicans* was still elusive. Our results demonstrated that C18 possesses antifungal activity not only against *C. albicans* but also on non-albicans species, especially against FLZ-resistant *C. tropicalis* at the concentration of 32 μg/ml. The drug-resistant *Candida* spp. turning sensitive to C18 also confirmed that the effect of C18 is not limited to a single *Candida* species and may represent another agent targeting these opportunistic fungi. Mechanistic insights revealed that C18 primarily target ROS generation and mitochondrial function in *C. albicans* that led to the damage of cell membrane and wall. In addition, C18 attenuated the pathogenicity of *C. albicans* and rescued 70% of *C. albicans*-infected *G. mellonella* larva.

In the recent years, emergence of some non-albicans *Candida* species, such as *C. tropicalis*, *C. glabrata*, *C. krusei*, *C. parapsilosis, C. auris*, etc., have dramatically increased (Whaley et al., 2016; Spivak and Hanson, 2018). The antifungal susceptibility profile might be different, so examination the antifungal activity of the peptide on clinical *Candida* isolates is necessary. C18 had antifungal susceptibility toward 4 strains of clinically FLZ-resistant *C. tropicalis*, with the lower MIC on FLZ-resistant strains than *C. albicans* reference (Table 1). Furthermore, the growth curve exhibited that C18 retarded *C. albicans* reaching to logarithmic phrase and 1 × MIC of C18 could reduce more than 99% fungi within 4 h from the time-kill kinetics (Figure 2). However, a small number of cells were not killed by C18 and proliferated after incubation for 4 h. We suspected these survival cells might become tolerant to the peptide when exposed to C18. According to recent reports, a heterogeneous behavior with respect to antibiotic survival called antibiotic persistence has been concerned. There is a small part of the clonal population (an average less than 1%) that are able to survive in bacteria or *Candida* spp. during exposure to the lethal concentrations of antibiotics (Wuyts et al., 2018; Gollan et al., 2019). These survival cells are called persisters and are killed at a slower rate than susceptible cells (Gollan et al., 2019). Furthermore, persisters have been initially detected in mature biofilms of *C. albicans* and in several other *Candida spp.* (Galdiero et al., 2020). In a way, tolerance and persistence are similar phenomena of increased survival in the presence of a lethal concentration of antibiotics (Balaban et al., 2019). We suspected that the very small amount of survival *C. albicans* cells during C18 exposure might be persisters.

Biofilm formation has been considered as a crucial virulence factor of *C. albicans* that is closely associated with its pathogenesis. Biofilm and hyphae growth are difficult to treat and require higher concentration of drug than planktonic cells, which contributes to the emergence of clinically resistant strains (Taff et al., 2013). Here, we found that C18 could inhibit the yeast-to-hypha transition (Figures 3A,B), which is key to biofilm maturation of *C. albicans*. Corresponding with the inverted microscope observations, the XTT reduction assays exhibited 32 μg/ml (1 × MIC) C18 and could inhibit more than 80% biofilm formation (Figure 3C), which may be attributed to inhibition of filamentation development (Park et al., 2021). This suggested that C18 can attenuate pathogenesis via inhibition of hyphal growth and biofilm formation. On the other hand, C18 displays unideal suppressant on preformed biofilm, with only about 50% preformed biofilm eliminated when exposed to 512 μg/ml (16 × MIC) C18 (Figure 3D). It has been reported that filamentous cells of mature biofilm are surrounded by an extracellular matrix contain proteins, carbohydrates, lipids, and nucleic acids (Lohse et al., 2018). The biofilm matrix functions as a protective physical barrier from environment, which may prevent C18 from eliminating preformed biofilm. The effect of C18 on biofilm matrix is unknown and further study is needed to explore the details. The antibiofilm results implied that C18 has potent inhibitory activity on biofilm formation and certain eradication effect on mature biofilm.

Antifungal activity of C18 on planktonic *C. albicans* combined with antibiofilm ability laid a favorable foundation for performing therapeutic effect of the peptide *in vivo*. In evaluating the potential antifungal effect of C18 *in vivo*, Galleria mellonlla larvae have proven to be a promising substitute of mammals (Glavis-Bloom et al., 2012). As a result of studying the survival rate of *C. albicans*-infected *G. mellonella* larvae by treatment with C18 in the i*n vivo* experiment, it was confirmed that C18 has low toxicity to *G. mellonella* larvae and exhibits a therapeutic effect on *C. albicans* infection *in vivo* (Figure 4), demonstrating that the pathogenicity of *C. albicans* was reduced. Meanwhile, the LD 50 (lethal dose of a drug for 50% of the population) of C18 toward the liver-derived HepG2 cell lines were found to be > 64 μg/ml (Peng et al., 2021). Wu et al. reported that some antifungal agent exhibited therapeutic effects on *C. albicans*-infected *G. mellonella*, which was closely related its antibiofilm activity (Wu et al., 2020). We predicted that C18 owns a weak toxicity to mammalian cells and *G. mellonella* larvae and reduced the morphogenesis of *C. albicans* that contributed to well therapeutic effects on *C. albicans*-infected larvae.

Fungal cell wall plays an essential role in the interaction with host cells, regulating processes such as adhesion or phagocytosis that are vital mediating infection (Arana et al., 2009). Most AMPs target the cell wall and membrane of fungi (Buda De Cesare et al., 2020; Tong et al., 2020; Yang et al., 2022). Defensins, cationic antimicrobial peptides, can insert in phospholipid bilayer of fungal cell walls, leading to disruption and subsequent death of the microorganisms (Yasui et al., 2017). Here, *C. albicans* treated with C18 exhibited certain deformities and pores in the cell morphology. Besides, *C. albicans* cells became bigger when exposed to C18 (Figure 5), which might be a state before integration caused by the change of osmotic stress of cell wall. Excessive degradation or reduction synthesis of cell wall components may result in loosening of cell wall, which in turn leads to swelling (Xu et al., 2014). The damaged cell wall causes osmotic disorders in the fungal cell, rupture of the cell membrane, and, consequently, abnormal exchange of cytoplasmic content and cell death (Fiołka et al., 2020). As previously reported, C18 can damage membrane of MRSA by altering membrane potential and increasing fluidity (Peng et al., 2021). Similarly, we also found that C18 could increase the membrane permeability and damage membrane integrity of *C. albicans* by PI uptake assays (Figure 6). Reportedly, membrane permeabilization is mostly associated with net positive charge of peptides (Seyedjavadi et al., 2019). Since C18 is positively charged (+ 5) and contains many hydrophobic residues, it was assumed that the increased membrane permeability of *C. albicans* induced by C18 might be ascribed to this peptide’s interaction with the negative-charged components (such as phosphatidylserine and phosphatidylinositol) of the fungal surface and therefore disrupt the membrane barrier. The results indicate that alteration of membrane permeability and cell wall caused by C18 might allow the passage of ions or large molecules entry into the cell and thus leading to cytoplasmic membrane dysfunction and cell death.

Reportedly, damage of membrane integrity has been associated with imbalance in the generation of ROS in yeast (Taff et al., 2013; Magill et al., 2014). Reactive oxygen species can easily produce polar lipid hydroperoxides, which is known to perturb the bilayer membrane structure and alter membrane properties (Evans et al., 2004). In the previous report, the parent peptide family’s member of C18, named Cecropin A, was found to induce cell death in human promyelocytic leukemia cells regulated by ROS signal mechanism (Cerón et al., 2010). In this work, by studying the result of ROS with DCFA-DA staining, the amount of ROS apparently increased in the presence of C18 (Figure 7A). Hence, ROS accumulation and, therefore, oxidative stress triggers a series of downstream cellular events that lead to disorganization of DNA and, therefore, cell death (Saibabu et al., 2020). Weak and dispersive DAPI staining observed in the cells with high level of ROS expression mirrored DNA damage (Figure 7A). On other hand, ROS accumulation can induce Ca^2 +^ release from endoplasmic reticulum (ER) or vacuole where the major source of Ca^2 +^ in *C. albicans* cytoplasm comes from Tao et al. (2016). Our findings represented that C18 promoted excess production cytosolic Ca^2 +^ labeled with Fluo-3/AM in a dose-dependent manner (Figure 7B). It seemed that excessive ROS generation driven cytosolic Ca^2 +^ accumulation is causative way to damage membrane permeability and cell death. In turn, elevating cytosolic Ca^2 +^ can perturbs mitochondrial Ca^2 +^ homeostasis, resulting in mitochondrial ROS generation (Fei et al., 2020). ROS and Ca^2 +^ signaling can be regarded as a bidirectional action because ROS can regulate cellular Ca^2 +^ signaling and Ca^2 +^ signaling is key to regulate mitochondria for ROS production (Oh et al., 2021). Interestingly, another parent peptide-Cecropin 3 was found to promote ROS overproduction and affected Ca^2 +^ transport when treated with rat cardiac muscle mitochondria in the research of Mitochondrial inactivation by cecropin 3 (Pavón et al., 2014). Therefore, excessive ROS generation and cytosolic Ca^2 +^ in *C. albicans* cells suggests that mitochondrial dysfunction of *C. albicans* has probably occurred after C18 treatment.

Mitochondrial dysfunction is known to influence drug susceptibilities and virulence traits of *C. albicans* (Lu et al., 2018; Verma et al., 2018). Several studies have demonstrated that AMPs targets mitochondria or ROS in different cellular systems (Lee et al., 2019; Liu et al., 2019). Mitochondria not only provide cells enough energy, but they also serve as a part of intracellular Ca^2 +^ stores and regulate intracellular Ca^2 +^ homeostasis and, most importantly, mediate cell death (Rui et al., 2010). When intracellular Ca^2 +^ generation increase, mitochondria will take in Ca^2 +^ from the cytoplasm to keep the intracellular Ca^2 +^ homeostasis (Dou et al., 2019). However, massive Ca^2 +^ in the mitochondria matrix promotes ROS production, which opens the membrane permeability transition pore (PTP) (Tian et al., 2017). The opening of the PTP results in mitochondrial dysfunction would disrupt △Ψ_m_ for ATP synthesis and trigger oxidative and anoxic cell death (Wu et al., 2009). For the effect of Cecropins on mitochondria, Cecropin A as a highly membrane-active peptide can affect mitochondrial coupling, respiration, and protein import associated with membrane potential (Hugosson et al., 1994). Thus, we assumed that ROS generation and Ca^2 +^ accumulation induced by C18 could affect △Ψ_m_, and it was confirmed that △Ψ_m_ loss was observed in the presence of C18, indicating membrane depolarization (Figure 8A). Cerón et al. (2010) also revealed that Cecropin A caused △Ψ_m_ collapse accelerating cell apoptosis in the process of Cecropin A-inducing caspase-independent cell death. In addition, ATP production primarily depends on oxidative phosphorylation in the mitochondria and is dynamically regulated by Ca^2 +^ levels in the mitochondrial matrix. For ATP assay results, the intracellular ATP content of *C. albicans* cells dramatically decreased after C18 treatment (Figure 8B), resulting in the abnormal homeostasis of many components in cells. Moreover, mitochondrial dysfunction might facilitate more ROS generation, exacerbating cells fate. These results suggested that C18 triggers mitochondrial dysfunction with the feature of △Ψ_m_ collapse and ATP depletion due to excessive intracellular Ca^2 +^ disturbing the balance between the Ca^2 +^ influx and export that leads to a progressive mitochondrial Ca^2 +^ uptake.

## Conclusion

**FIGURE 9:**
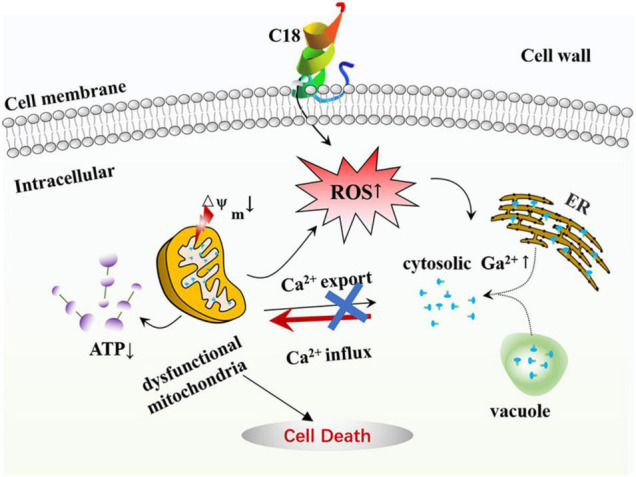
The mode of C18 against *Candida albicans*. C18 has an significant inhibitory efficacy on *C. albicans*, such as abnormal morphogenesis, cell wall damage, and alteration of membrane permeability, resulting in a series of destructive effects in cytoplasm. Causally, C18 plays an essential role in ROS generation and intracellular Ca^2 +^ increase, which contributes to dysfunctional mitochondria in form of △Ψ_m_ collapse and ATP depletion. In short, C18 can trigger mitochondrial dysfunction driven by excessive ROS production and Ca^2 +^ accumulation, leading to the cell death.

In this study, C18 shows a significant antifungal activity against *C. albicans* and FLZ-resistant *C. tropicalis*, and also improves survival rate of *C. albicans*-infected larvae *in vivo*. As outlined in Figure 9, we demonstrated that C18 can damage the cell wall and alter cell membrane permeability, attenuating virulence in *C. albicans*. Furthermore, the peptide triggers dysfunction mitochondria with the feature of △Ψ_m_ collapse and ATP depletion induced by ROS generation and intracellular Ca^2 +^ accumulation, leading to cell death. Our data has shown considerable promising antifungal possibility of C18 and is valuable to draw attention for further pharmacodynamic investigations in animal models. Further studies related to drug combination could be an attractive approach to increase its potency. Our findings highlight new strategies for the treatment of candidiasis and reveals the possible molecular mechanisms of inhibitory *C. albicans*.

## Data Availability Statement

The original contributions presented in the study are included in the article/Supplementary Material, further inquiries can be directed to the corresponding author.

## Author Contributions

C-QS initiated the study and drafted the manuscript. C-QS and GG designed the experiments and wrote the manuscript. C-QS, L-BY, R-YT, L-JZ, Z-QT, and M-JH performed the experiments and interpreted the data. JP, Z-LJ, and L-XZ contributed materials and analysis tools. All authors have read the manuscripts and approved the submitted version.

## Conflict of Interest

The authors declare that the research was conducted in the absence of any commercial or financial relationships that could be construed as a potential conflict of interest.

## Publisher’s Note

All claims expressed in this article are solely those of the authors and do not necessarily represent those of their affiliated organizations, or those of the publisher, the editors and the reviewers. Any product that may be evaluated in this article, or claim that may be made by its manufacturer, is not guaranteed or endorsed by the publisher.

## Footnotes

1
https://heliquest.ipmc.cnrs.fr/cgi-bin/ComputParams.py

2
https://zhanggroup.org/I-TASSER/
