# Supplementary material for: A Cecropin-4 Derived Peptide C18 Inhibits Candida albicans by Disturbing Mitochondrial Function
Source: Front Microbiol. 2022 Apr 19;13:872322. doi: 10.3389/fmicb.2022.872322 (PMC9075107; doi:10.3389/fmicb.2022.872322)
Supplement: Supplementary file 1 [file Data_Sheet_1.docx]

Supplementary Material

**Figure S1.** Mass spectrum of the synthetic peptide C18. Mass spectrometry report showed its molecular weight 2181.73 Da and purity were, 95.53%.

Mass Spectrometry Report

| Sample Description | | Instrument |  | Agilent-6125B | |
| --- | --- | --- | --- | --- | --- |
| Analyzed date: | 2020-11-06 | Probe: | ESI | Probe Bias: | ＋4.5kv |
| Analyst: | YU | Nebulizer Gas Flow: | 1.5L/min | Detector: | 1.5kv |
| Sample: | “C18 LR-16 | CDL: | -20.0v | T. Flow: | 0.2ml/min |
| M.W.: | 2181.73 | CDL Temp.: | 250 | ^o^C | B. Conc.: |
| Lot. No.: | P201102-CL826461 | Block Temp.: | 200 | ^o^C |  |

**Figure S2.** High performance liquid chromatography **(**HPLC) report of the synthetic peptide C18.

**HPLC REPORT**

Product Name: “C18 LR-16

Instrument No: 0200194

Lot No :P201102-CL826461

Column :4.6*250mm C18

Solvent A :0.1%Trifluoroacetic in 100% Acetonitrile

Solvent B :0.1%Trifluoroacetic in 100% Water

Gradient : A B

0.01min 27% 73%

25min 52% 48%

25.01min 100% 0%

30.0min STOP

Flow rate :1.0ml/min

Wavelength :220nm

Volume: 10µl

────────────────────────────

Rank Time Name Conc. Area Height

────────────────────────────

1 11.250 0.4895 16811 2497

2 11.421 95.52 3279958 311860

3 11.770 3.995 137191 8275

────────────────────────────

Total 100 3433960 322632
